# Microbial diversity in the deep-subsurface hydrothermal aquifer feeding the giant gypsum crystal-bearing Naica Mine, Mexico

**DOI:** 10.3389/fmicb.2013.00037

**Published:** 2013-03-06

**Authors:** Marie Ragon, Alexander E. S. Van Driessche, Juan M. García-Ruíz, David Moreira, Purificación López-García

**Affiliations:** ^1^Unité d’Ecologie, Systématique et Evolution, CNRS UMR 8079, Université Paris-SudOrsay, France; ^2^Laboratorio de Estudios Cristalográficos, Instituto Andaluz de Ciencias de la Tierra, Consejo Superior de Investigaciones CientíficasUniversidad Granada, Granada, Spain

**Keywords:** aerobic ammonium oxidation, Candidate Division OP3, GC content, hydrothermal, Thaumarchaeota, thermophile, Thermoplasmatales

## Abstract

The Naica Mine in northern Mexico is famous for its giant gypsum crystals, which may reach up to 11 m long and contain fluid inclusions that might have captured microorganisms during their formation. These crystals formed under particularly stable geochemical conditions in cavities filled by low salinity hydrothermal water at 54–58°C. We have explored the microbial diversity associated to these deep, saline hydrothermal waters collected in the deepest (ca. 700–760 m) mineshafts by amplifying, cloning and sequencing small-subunit ribosomal RNA genes using primers specific for archaea, bacteria, and eukaryotes. Eukaryotes were not detectable in the samples and the prokaryotic diversity identified was very low. Two archaeal operational taxonomic units (OTUs) were detected in one sample. They clustered with, respectively, basal Thaumarchaeota lineages and with a large clade of environmental sequences branching at the base of the Thermoplasmatales within the Euryarchaeota. Bacterial sequences belonged to the Candidate Division OP3, Firmicutes and the Alpha- and Beta-proteobacteria. Most of the lineages detected appear autochthonous to the Naica system, since they had as closest representatives environmental sequences retrieved from deep sediments or the deep subsurface. In addition, the high GC content of 16S rRNA gene sequences belonging to the archaea and to some OP3 OTUs suggests that at least these lineages are thermophilic. Attempts to amplify diagnostic functional genes for methanogenesis (*mcrA*) and sulfate reduction (*dsrAB*) were unsuccessful, suggesting that those activities, if present, are not important in the aquifer. By contrast, genes encoding archaeal ammonium monooxygenase (*AamoA*) were amplified, suggesting that Naica Thaumarchaeota are involved in nitrification. These organisms are likely thermophilic chemolithoautotrophs adapted to thrive in an extremely energy-limited environment.

## INTRODUCTION

At the onset of the 1980s, the detection of living microorganisms in deep sediment cores revealed the occurrence of a hitherto unsuspected but vast subsurface ecosystem associated to deep-sea sediments as well as continental and oceanic crusts (Ghiorse and Wilson, 1988; Gold, 1992; White et al., 1998; Whitman et al., 1998; Pedersen, 2000). Since then, the number of microbial diversity surveys on the deep subsurface has multiplied, especially in the subseafloor, benefiting from initiatives such as the International Ocean Drilling Program (IODP^1^). As data accumulate, initial quantitative assessments predicting that over 50% of all prokaryotic cells occur in the subsurface (Whitman et al., 1998) have been seriously challenged (Jorgensen, 2012; Kallmeyer et al., 2012). This illustrates that, despite increasing efforts, the diversity, extent, and function of the deep biosphere remain largely unknown.

This limited knowledge is due to a variety of factors including the inherent difficulty of sampling at progressively higher underground depths, the problem of external microbial contamination, and the refractory nature of many subsurface microbes that are dormant or living at extremely low metabolic rates (White et al., 1998; Teske, 2005). Indeed, although the deep subseafloor constitutes the largest biotope on Earth, it seemingly possesses the lowest metabolic rates. The deep biosphere is also highly heterogeneous. Hot spots of microbial activity occur whenever chemical energy derived from redox reactions involving organic matter or inorganic electron donors (hydrogen, methane, hydrogen sulfide, or iron) are available at sedimentary and/or geochemical interfaces (Chapelle et al., 2002; Parkes et al., 2005; Jorgensen and Boetius, 2007). Subseafloor sediments are rich in organic matter as compared to rocky environments and, thus, sustain relatively diverse microorganisms, some of which degrade aromatic and other recalcitrant compounds (Fredrickson et al., 1995). Nonetheless, the microbial activities that dominate in these settings are methanogenesis and sulfate reduction, which constitute the terminal steps in the degradation of organics in the biogeochemical carbon cycle (Newberry et al., 2004; Webster et al., 2006; Fry et al., 2008). Active microorganisms have been detected in marine subseafloor sediments down to depths of 1,626 m below the sea floor at the Newfoundland margin, which corresponds to the oldest (111 million years old), and potentially hottest (~100°C) marine sediments investigated (Roussel et al., 2008). Deep oil/petroleum reservoirs also sustain active microbial communities and several of their members are able to degrade long hydrocarbon chains and other complex organics (Rueter et al., 1994; Head et al., 2003; Kim and Crowley, 2007).

However, most of the deep subsurface, especially the bed rock, harbors organisms living under extreme energy limitation. Under these conditions, microorganisms are likely inactive or display exceedingly low metabolic activities (Jorgensen and D’Hondt, 2006; Jorgensen and Boetius, 2007). The detection of subsurface microorganisms may be hampered by such low metabolic rates (Teske, 2005) but also by the occurrence of divergent phylogenetic lineages, since general primers used to amplify marker ribosomal RNA genes may fail to amplify genes from such divergent clades, a problem that may typically affect the archaea (Teske and Sorensen, 2008). The volcanic oceanic crust has also been less explored due to the challenging sampling conditions and the needs for specific equipment (Edwards et al., 2011). Nonetheless, microbial communities have been recently studied in subseafloor basement fluids as deep as 2,667 m on the eastern flank of the Juan de Fuca Ridge in the Pacific (Jungbluth et al., 2012). While very deep rock communities in, for instance, granite or basalts, are much poorer than sediment communities due to the extreme nutrient depletion, organisms able to degrade hydrocarbons that might originate from serpentinization have been detected in very deep ocean gabbro (Mason et al., 2010), opening an intriguing new window for life in such ecosystems.

The microbiology of the deep continental crust is even less well explored than that of the oceanic crust. Among the investigated sites, often from an applied perspective, are deep aquifers (Kimura et al., 2005), potential sites for the storage of radioactive waste (Stroes-Gascoyne and West, 1997) or gas (Basso et al., 2009), and deep mines (Onstott et al., 2003; Sahl et al., 2008; Rastogi et al., 2010). One of the deepest sites studied is the Archaean metabasalt at 2.825 km below the land surface in the Mponeng gold mine, South Africa, where sulfate reducers belonging to the Firmicutes appeared to be sustained by geologically produced sulfate and hydrogen (Lin et al., 2006b). Deep mines offer a relatively easy access to the continental subsurface biosphere, and allow for sampling in very different geological settings.

In this work, we have explored the microbial diversity associated to a deep, saline hydrothermal aquifer by sampling water springing at ca. 60°C in the deepest (ca. 700 m) shafts of the Naica Mine, Mexico. Naica, one of the most important lead and silver deposits in the world, is located in a semi-desertic region but, during the raining season, temporary lagoons and flooding zones parallel to the regional fault system may recharge the hydrothermal aquifer (Villasuso, 2002). The Naica area is under a mild thermal anomaly and water springing in the mine galleries has a temperature ranging from 50 to 60°C (Garcia-Ruiz et al., 2007). The unique geochemical conditions of the mine have led to the formation, at very low calcium-sulfate supersaturation, of giant gypsum crystals (Erwood et al., 1979; Garcia-Ruiz et al., 2007; Van Driessche et al., 2011). Some of these giant crystals contain fluid inclusions that might have captured microorganisms during their formation. The description of current microbial communities associated to the subsurface hydrothermal water where these crystals formed is a pre-requisite to predict the type of microorganisms that may have been trapped in fluid inclusions and a control to distinguish potential contaminants during the analysis of such small fluid inclusions.

## MATERIALS AND METHODS

### SAMPLING AND DNA PURIFICATION

**FIGURE 1:**
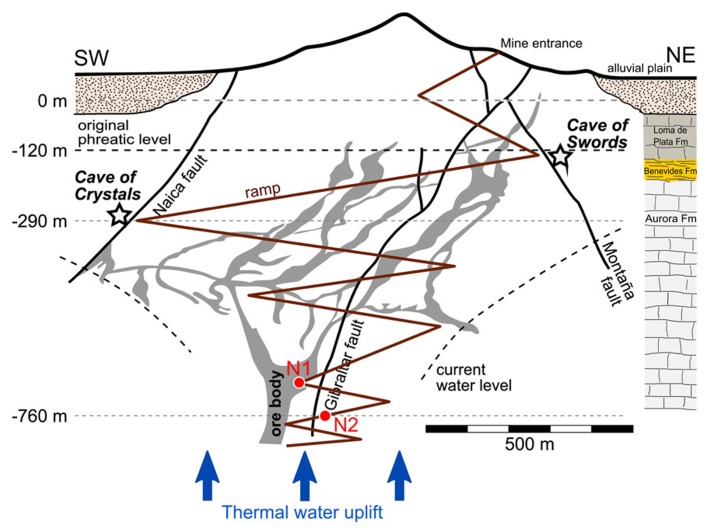
**Hot spring sampling sites in the Naica Mine.** Water sampling sites N1 and N2 are indicated in a not-to-scale hydrogeological sketch of the Naica deposit and associated caves. The horizontal groundwater level marks the position of the phreatic level immediately before the discovery of Cueva de la Espadas in 1910. The stratigraphic sequence on the right reports the lithology of a 1,150-m deep drillcore of the area (Garofalo et al., 2010). The figure was adapted from (Garcia-Ruiz et al., 2007) and (Forti and Sanna, 2010).

The Naica Mine is located 112 km southeast of Chihuahua City in northern Mexico (27°51′3^′′^N; 105°29′47^′′^W). A further description of the geological and hydrological setting of the mine is provided as Appendix. Water samples N1 and N2 were collected in the deepest excavated Naica mineshafts at ca. 700 and 760 m depth (**Figure 1**). Having been abandoned and partially inundated, these sites are seldom visited by miners, which limits potential contamination brought from the exterior or from upper layers of the mine. The air temperature was ca. 40°C and the humidity ca. 90%. The water vigorously sprang from fractures in the rock wall. The water temperature measured *in situ* in N1 and N2 was of ca. 60°C and the pH of 7.5. Chemical analyses of the aquifer water have been carried out from 1976 to 2003, showing a rather stable composition through time. The water is highly enriched in calcium (~600 mg/l) and sulfate (~2 g/l) but contains also 100 mg/l Na, 24 mg/l Cl, and 120 mg/l Mg as major components (see Table DR4 in Data Repository Item; Garcia-Ruiz et al., 2007). Two water samples of 20 l each were collected from two different hot springs (N1 and N2) in clean plastic containers thoroughly rinsed with the same hot spring water. The containers were immediately transported to the nearest laboratory and the water filtered directly through GTTP^TM^ isopore filters (Millipore, MA, USA) of 0.22 μm-pore diameter. Filters were fixed *in situ* in 96% ethanol and stored at -20°C until DNA was purified. For DNA extraction, the ethanol was removed from the filters by evaporation. The remaining ethanol was filtered through a small 0.22 μm-pore size filter to retain detached microbial biomass. Filters from each sample were cut in small fragments, which were immediately rehydrated in the initial resuspension buffer of the PowerSoil DNA isolation kit (Mo Bio, Laboratories Inc., CA, USA). DNA was purified then using the PowerSoil DNA isolation kit following the instructions of the manufacturer. DNA was eluted in 60 μl of Tris–HCl, pH 8 and conserved at -20°C.

### PCR AMPLIFICATION, CLONING, AND SEQUENCING

Small-subunit ribosomal RNA genes were amplified by polymerase chain reaction (PCR) using specific primers for each domain of life. For bacteria, we used the primers 27F (AGAGTTTGATCCTGGCTCAG) and 23S-1R (GGGTTTCCCCATTCGGAAATC), which also amplify the adjacent internal transcribed spacer (ITS) region. Semi-nested reactions were subsequently carried out with 27F and the reverse prokaryotic primer 1492R (GGTTACCTTGTTACGACTT). Archaeal 16S rRNA genes were initially amplified using the forward archaea-specific primer 21FQ (GGGCGGGCTTCCGGTTGATCCTGCCGGA) and 1492R. Subsequent semi-nested amplifications were carried out with the internal forward primer Ar109 (AC(G/T)GCTGCTCAGTAACACGT)(N1-4A), W36 (TCCAGGCCCTACGGGG) (N1-5A), and ANMEF (GGCTCAGTAACACGTGGA) (N1-6A). Attempts to amplify eukaryotic 18S rRNA genes were carried out using the specific primers 82F (GAAACTGCGAATGGCTC) and 1520R (CYGCAGGTTCACCTAC) followed by semi-nested PCRs with 1498R (CACCTACGGAAACCTTGTTA). PCR reactions were carried out in 25 μl of reaction buffer, containing 1.5 μl of the eluted DNA, 1.5 mM MgCl_2_, dNTPs (10 nmol each), 20 pmol of each primer, and 0.2 U Taq platinum DNA polymerase (Invitrogen). PCR reactions were performed under the following conditions: 35 cycles (denaturation at 94°C for 15 s, annealing at 55°C for 30 s, extension at 72°C for 2 min) preceded by 2 min denaturation at 94°C, and followed by 7 min extension at 72°C. Negative controls were carried out systematically for each PCR amplification experiment; all were negative. In addition, to identify and eliminate potential contaminant sequences introduced during the manipulation in the laboratory of samples with extremely low biomass, e.g., contaminants associated to the DNA extraction kit and process, we had previously built a database of potential contaminants (Gerard et al., 2009). Naica sequences were not closely related to them. Attempts to amplify *dsrAB* genes encoding the dissimilatory sulfite reductase diagnostic of sulfate reducers and *mcrA* genes encoding the methyl coenzyme M reductase characteristic of methanogenic archaea were done using, respectively, the specific primers DSR-1F (ACSCACTGGAAGCACG) + DSR-4R (GTG TAG CAG TTA CCG CA) (Perez-Jimenez et al., 2001) and ME1F (CGMATGCARATHGGWATGTC) + ME2R (TCATKGCRTAGTTDGGRTAGT) (Inagaki et al., 2004). Archaeal *amoA* genes encoding the ammonium monooxygenase were amplified with primers Arch-amoAF (STAATGGTCTGGCTTAGACG) and Arch-amoAR (GCGGCCATCCATCTGTATGT) (Francis et al., 2005). To amplify protein genes an annealing-temperature gradient of 47–55°C was used to maximize the chances of amplification. Clone libraries were constructed using the TopoTA cloning kit (Invitrogen, Carlsbad, CA, USA) according to the manufacturer’s instructions. Clone inserts were PCR-amplified using flanking vector primers, and inserts of expected size were partially sequenced (Beckman Coulter Genomics, Takeley, UK) with 1492R yielding sequences of 800–1,000 bp. A total of 91 archaeal and 266 bacterial high quality sequences were retained for this study. Potential chimeric sequences were identified manually by comparing BLAST (Basic Local Alignment Search Tool) results from several portions of the full-length sequence. Operational taxonomic units (OTUs) were defined as groups of 16S rRNA gene sequences sharing ≥98% identity. Several representative 16S rRNA clones of the different OTUs were nearly fully sequenced by using forward primers. Complete sequences were assembled using Code Aligner (CodonCode Corporation^2^) prior to phylogenetic analyses.

### PHYLOGENETIC ANALYSES

Environmental 16S rRNA gene sequences from the Naica Mine were compared with sequences in the database GenBank^3^ by BLAST (Altschul et al., 1997). We retrieved the closest sequences found in the database and included them in an alignment containing also sequences from the closest cultivated members and some representative sequences of the major taxa found. Sequences were aligned using MUSCLE (MUltiple Sequence Comparison by Log-Expectation; Edgar, 2004). Ambiguously aligned positions and gaps were eliminated using Gblocks (Castresana, 2000). A total of 1,079 and 846 unambiguously aligned nucleotide positions were retained for, respectively, archaeal and bacterial sequences in order to carry out subsequent phylogenetic analyses. The resulting sequence alignments were used as input to build phylogenetic trees by approximate maximum likelihood using Fasttree (Price et al., 2010) with a general time reversible (GTR) model of sequence evolution, and taking among-site rate variation into account by using a four-category discrete approximation of a γ distribution. ML bootstrap proportions were inferred using 1,000 replicates. For protein (amoA) phylogenetic analyses, amino acid alignments were obtained with MUSCLE and trimmed using Gblocks. The ML phylogenetic tree was reconstructed using Treefinder (Jobb et al., 2004) with the WAG-γ model of sequence evolution. Phylogenetic trees were visualized using the program FIGTREE^4^. The sequences reported in this study have been deposited in GenBank with accession numbers KC481372-KC481399.

## RESULTS AND DISCUSSION

### MICROBIAL DIVERSITY IN NAICA DEEP HYDROTHERMAL WATER

**FIGURE 2:**
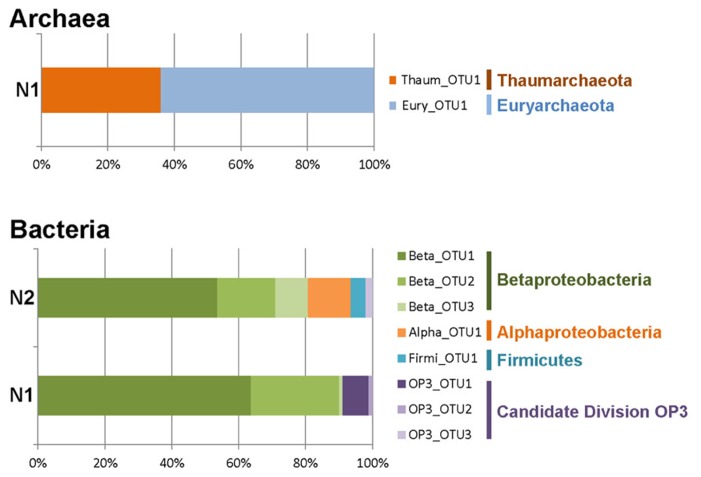
**Relative proportions of archaeal and bacterial operational taxonomic units (OTUs) distributed in major high-rank taxa from Naica deep hot springs.** Archaea were only detected in sample N1. Eukaryotes were not detected in any of the two samples.

We tried to amplify archaeal, bacterial, and eukaryotic 16S/18S rRNA genes in the samples collected in two of the deepest accessible fracture-associated springs in the Naica mineshafts at approximately 700 and 760 m below the entrance level (**Figure 1**). However, despite several attempts, including nested PCR experiments, we failed to amplify eukaryotic 18S rRNA genes. Similarly, we failed to amplify archaea from N2. By contrast, we detected archaea belonging to the Thaumarchaeota and the Euryarchaeota in N1 (**Figure 2**). Nested PCR reactions were also required to amplify bacterial 16S rRNA genes, indicating that microbial biomass in this thermal water is very low. Only two high rank-bacterial taxa were detected in both samples, Betaproteobacteria, which represented between 80% (N2) and 90% (N1) of the 16S rRNA gene sequences analyzed, and the Candidate Division OP3, which represented 2% (N2) and 10% (N1) of the clone sequences. N2 displayed a higher bacterial diversity, with a few additional representatives of Firmicutes and Alphaproteobacteria accounting for up to 18% of the sequences (**Figure 2**).

The low prokaryotic diversity was not only observed at the level of high-rank taxa but also within taxa. Thus, very few OTUs (defined at ≥98% sequence similarity level) were detected. Thaumarchaeota, Euryarchaeota, Alphaproteobacteria, and Firmicutes were represented by a single OTU, while up to three OTUs were detected within the Beta-proteobacteria and the Candidate Division OP3 (**Figure 2**). Nonetheless, the different OTUs were not singletons, but encompassed several closely related sequences, sometimes detected in both samples N1 and N2.

**FIGURE 3:**
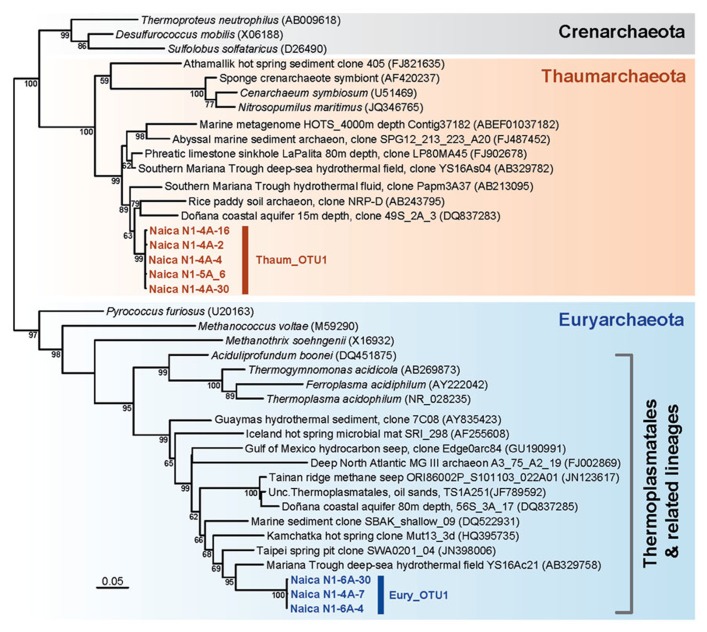
**Maximum likelihood phylogenetic tree of archaeal 16S rRNA genes retrieved from Naica deep hot springs.** The tree was reconstructed using 1,079 positions. Sequences obtained in this work are shown in color. Accession numbers of sequences retrieved from GenBank are given between brackets. Only bootstrap values higher than 50% are given at nodes. The scale bar represents the number of substitutions per a unit branch length.

The two detected archaeal OTUs formed clades with other environmental sequences, but were very far from sequences of cultured species. The Thaumarchaeota Thaum_OTU1 clustered with a group of sequences retrieved mostly from deep-sea waters, sediments, and fluids, including sequences from crustal fluids in back-arc hydrothermal fields of the southern Mariana Trough (Kato et al., 2009; **Figure 3**). Related sequences came from boreholes in other Mexican regions and from subsurface coastal aquifers (López-Archilla et al., 2007). Similarly, the Euryarchaeota Eury_OTU1 belonged to a large clade consisting exclusively of environmental sequences branching as a sister group to the Thermoplasmatales. Again the closest neighbors were sequences retrieved from deep-sea hydrothermal fields, continental hot springs or pits (**Figure 3**). The Thaumarchaeota or Group I archaea constitute one of the most diversified archaeal phyla, being abundant in deep-sea plankton and soils, but also frequently associated to hot springs and the deep subsurface (Chandler et al., 1998; Rastogi et al., 2009). While many of the marine and soil mesophilic Thaumarchaeota may be nitrifiers, aerobically oxidizing ammonia to nitrite, the metabolism of the deeper Thaumarchaeota branches to which the Naica OTU belongs, remains unknown (Pester et al., 2011). The Thermoplasmatales are moderately thermoacidophilic archaea frequent in hot springs. The related lineages to which the Naica sequences resemble have been identified in deep-sea sediments and also in the deep subsurface (Lin et al., 2006a; Kato et al., 2009; Zhang et al., 2010). This suggests that both archaeal OTUs are autochthonous lineages in the Naica hydrothermal springs.

**FIGURE 4:**
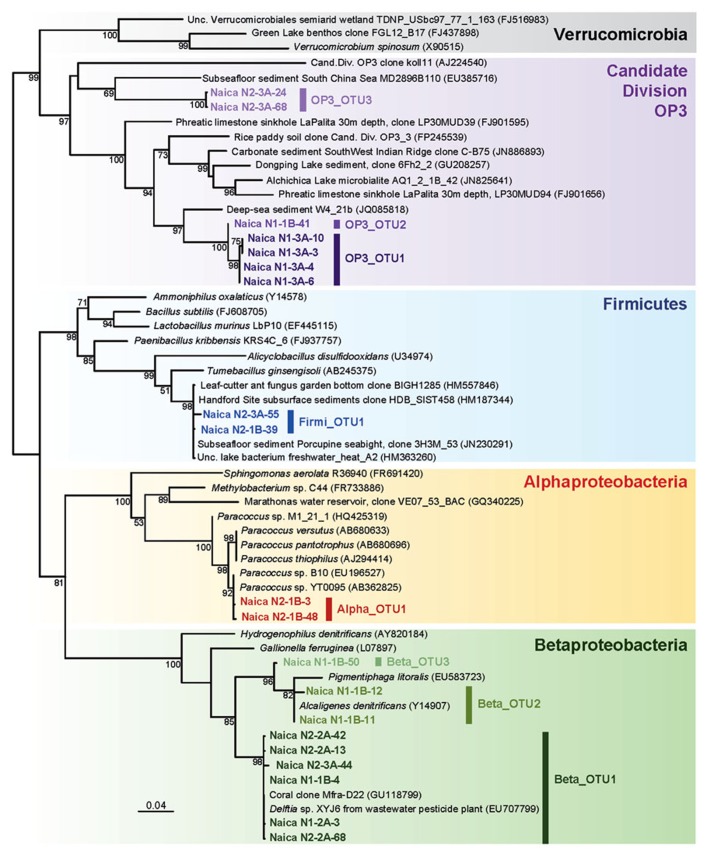
**Maximum likelihood tree of bacterial 16S rRNA genes retrieved from Naica deep hot springs.** The tree was reconstructed using 846 positions. Sequences obtained in this work are shown in color. Accession numbers of sequences retrieved from GenBank are given between brackets. Only bootstrap values higher than 50% are given at nodes. The scale bar represents the number of substitutions per a unit branch length.

Bacteria were dominated by the Betaproteobacteria, and in particular by the Beta_OTU1, which represented between 50 and 65% of the sequences retrieved in N1 and N2. Naica sequences were identical or nearly identical to *Delftia* sp. XYJ6, a strain isolated from wastewater and being able to degrade aniline (Yan et al., 2011) and to an environmental sequence obtained from a coral (**Figure 4**). By contrast the Beta_OTU2 is most closely related to *Alcaligenes denitrificans*, which can be an opportunistic pathogen. This OTU might therefore represent an external contaminant.

The remaining bacterial OTUs appear also to be autochthonous to the Naica system. The alphaproteobacterial OTU detected in N2 ascribed to the genus *Paracoccus*, encompassing bacteria often associated to soils but which can be isolated from the deep subseafloor (Kobayashi et al., 2008). Likewise, the Firmicutes sequences (Firmi_OTU1) were nearly 100% identical to sequences retrieved from a subsurface aquifer at the Hanford Site (USA; Lin et al., 2012) and also to sequences retrieved from deeply buried coral carbonates and sediment at Porcupine Seabight (site U1317 Hole A; **Figure 4**). Finally, the three OTUs ascribing to the Candidate Division OP3 are most likely indigenous as well. So far, there is no cultured member of this taxon, but their 16S rRNA sequences have been identified in a variety of ecosystems such as marine sediments, hypersaline deep-sea, freshwater lakes, aquifers, flooded paddy soils, and methanogenic bioreactors (Madrid et al., 2001; López-Archilla et al., 2007; Glockner et al., 2010). The fact that many of these environments are anoxic and the identification of OP3 genes potentially involved in anaerobic respiration in metagenomic libraries suggest that many OP3 bacteria are anaerobes (Glockner et al., 2010). Very recently, some OP3 members have been shown to be magnetotactic bacteria (Kolinko et al., 2012). They have been notably detected in fracture-derived groundwater in a deep gold mine of South Africa (Lin et al., 2006a). The Naica OP3 OTUs are divergent and have as closest relatives, although relatively distant, environmental sequences retrieved from deep-sea sediments or the subseafloor (**Figure 4**).

## LIKELY THERMOPHILIC LIFESTYLE UNDER EXTREME ENERGY LIMITATION

The microbial diversity identified in the Naica hydrothermal water samples N1 and N2 was very low, being only comparable to that found in strongly energy-limited areas of the subsurface (Jorgensen and Boetius, 2007). Even samples from other deep-sea mines seemed to host a larger microbial diversity (Onstott et al., 2003; Lin et al., 2006b; Sahl et al., 2008; Rastogi et al., 2010), although the introduction of external contaminants by the mining activity or during the processing of extremely low biomass samples remains a general risk in deep-subsurface studies. In our case, most of the OTUs identified had as closest relatives other environmental sequences coming from deep sediments or subseafloor environments, suggesting that many of the microorganisms detected are indeed autochthonous to the Naica system. This is particularly clear for the archaea and bacteria of the Candidate Division OP3, and possibly for the Firmicutes OTU identified. With the exception of Beta_OTU2, which may be a human-related contaminant, the remaining Alpha- and Beta-proteobacterial sequences identified in Naica samples may be also indigenous. However, the possibility that they are associated to soil or dust particles that have been introduced in the shaft cannot be completely ruled out. Given the low prokaryotic diversity of the system and its extreme conditions, especially the relatively high temperatures and the high nutrient depletion (oligotrophy), the absence of detectable eukaryotes in the Naica hot springs was not surprising.

**FIGURE 5:**
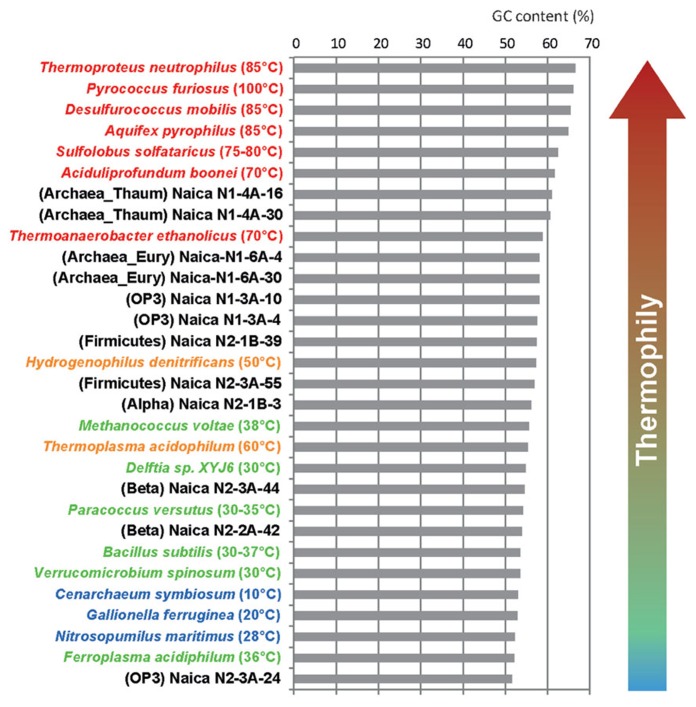
**Comparative histogram showing the GC content of the same 16S rRNA region in Naica clones and in a wide range of microbial species growing at different temperatures**.

The above observations were further confirmed by the GC content of the retrieved sequences, which suggests that the archaeal and at least two OTUs of the Candidate Division OP3 correspond to thermophilic organisms. Although GC content varies along the genome and across phylogenetic lineages, it is well known that thermophiles and, most especially, hyperthermophiles increase the GC content of their rRNA molecules to cope with high temperatures (Groussin and Gouy, 2011). As can be seen in **Figure 5**, the GC content of Naica archaea, OP3 OTUs 1, and 2 and the Firmicutes fall among that of organisms growing optimally at temperatures between 50 and 70°C. This suggests that these organisms are indeed thermophiles. The Thaumarchaeota, with over 60% GC at their 16S rRNA genes might be even extreme thermophiles and be able to grow in even hotter areas of the aquifer (~60–70°C). By contrast, little can be said about the optimal growth temperature of the remaining OTUs that display lower GC content in their rRNA sequences. They could be mesophiles or they might be moderate thermophiles growing well at temperatures around 50–55°C in the Naica system. Intriguingly, the sequences with the lowest GC content were those of the Candidate Division OP3 OTU3 (**Figure 5**), which is most certainly autochthonous to Naica. This might indicate an overall low GC content in the genome of these organisms.

**FIGURE 6:**
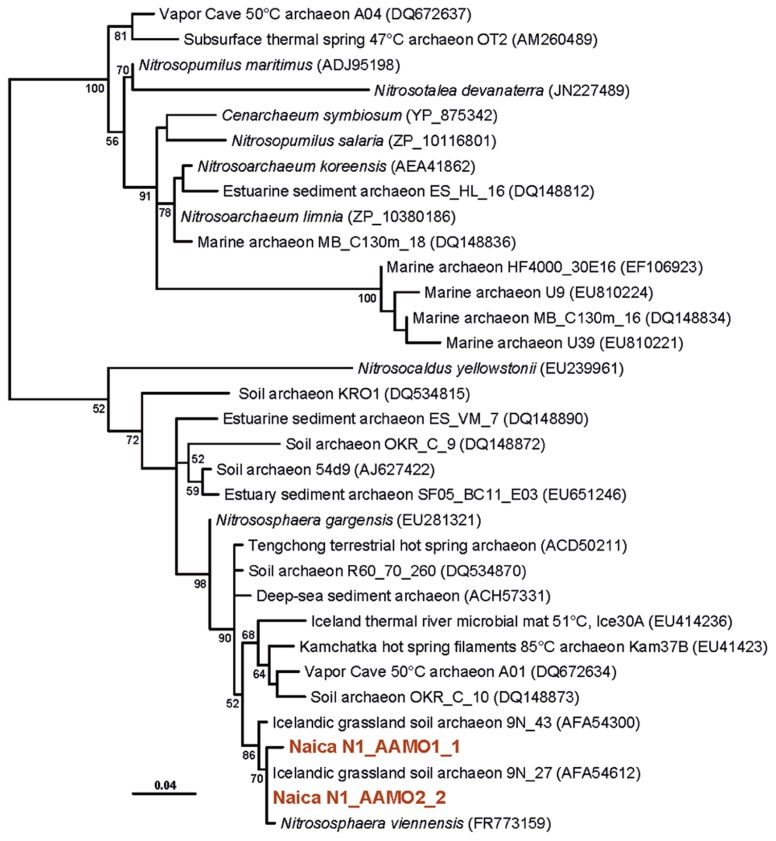
**Maximum likelihood tree of archaeal amoA genes retrieved from Naica deep hot springs.** The tree was reconstructed using 203 amino acid positions. Sequences obtained in this work are shown in color. Accession numbers of sequences retrieved from GenBank are given between brackets. Only bootstrap values higher than 50% are given at nodes. The scale bar represents the number of substitutions per a unit branch length.

With the exception of the thermophilic character of microorganisms thriving in Naica waters, little can be said about their metabolic potential from their 16S rRNA genes. Therefore, we aimed at providing some metabolic information about the Naica community by amplifying genes involved in specific metabolic pathways. In the case of archaea, many Thaumarchaeota are known to be nitrifiers, oxidizing ammonia aerobically to nitrite, although the presence of archaeal *amo* genes does not necessarily correlate with the basal branching groups, leaving the question open for these basal Thaumarchaeota (Pester et al., 2011). We succeeded in amplifying archaeal *amoA* genes, which branched together with *amoA* genes coming from soils and hot springs forming a clade distinct from that of marine planktonic archaea (**Figure 6**). This strongly suggests that these Naica archaea are thermophilic nitrifiers that, given the highly oligotrophic conditions, are likely chemolithoautotrophic, as other members of the group (Pester et al., 2011).

Methanogenic archaea and sulfate-reducing bacteria are often detected in subsurface environments (Jorgensen and Boetius, 2007; Fry et al., 2008; Roussel et al., 2008). However, archaeal sequences belonging to classical methanogenic Euryarchaeota were not observed in Naica. Nonetheless, very recently, the occurrence of methanogenesis has been discovered in organisms branching at the base of the Thermoplasmatales (Paul et al., 2012). Although our Naica sequences are not very closely related to these new “Methanoplasmatales,” they remain related to deep-sea sequences forming a cluster with them (**Figure 3**; Paul et al., 2012). However, we failed to amplify *mcrA* genes, used as a marker for methanogenesis, suggesting that Eury_OTU2 are not methanogens and gain energy by an unknown mechanism.

In principle, the presence of sulfate-reducing bacteria could be expected in Naica since its hydrothermal waters are highly enriched in sulfate, allowing for the formation of massive gypsum crystals at a delicate super-saturation balance between anhydrite and gypsum (Garcia-Ruiz et al., 2007; Van Driessche et al., 2011; Krueger et al., 2013). However, members of the sulfate-reducing Deltaproteobacteria were not identified. Sulfate reducers are also found within the Firmicutes, but the Naica OTU is not closely related to known Gram-positive sulfate reducers (**Figure 4**), suggesting that they use another type of metabolism. We failed to amplify *dsrA* genes encoding the dissimilatory sulfite reductase, which further reinforces the idea that Naica-associated microbial communities do not (or not dominantly) carry out sulfate reduction. At any rate, for sulfate reduction to occur, either organic matter or inorganic electron donors, typically H_2_, must be present to fuel the redox reaction. However, both appear to be extremely low in the aquifer. Conversely, appreciable amounts of H_2_S, the resulting product of sulfate reduction, are not detectable in Naica (unpublished observations). All these observations suggest that sulfate reduction, if it exists, is not a dominant metabolism in the microbiota of the Naica hydrothermal water despite an overwhelming availability of sulfate. This highlights the importance of having access to redox interfaces for life and implies that microorganisms thriving in the deep-subsurface thermal waters of the Naica system are among the most oligotrophic and energy-challenged communities explored to date.

Given the low biomass and diversity associated to the Naica thermal water, the possibility that microorganisms were entrapped in fluid inclusions in the massive crystals that slowly formed in these saline hot waters is small. Nevertheless, the present description offers a list of potential bona-fide candidate lineages to be captured in the fluid inclusions. Whether their macromolecules, especially their DNA, have escaped thermal degradation upon metabolic exhaustion in the fluid entrapments and are still detectable should be the subject of future studies.

## Conflict of Interest Statement

The authors declare that the research was conducted in the absence of any commercial or financial relationships that could be construed as a potential conflict of interest.

## Footnotes

1 http://www.iodp.org/

2 http://www.codoncode.com

3 http://www.ncbi.nlm.nih.gov/

4 http://tree.bio.ed.ac.uk/software/figtree/

## APPENDIX

### HYDROLOGICAL AND GEOLOGICAL SETTING OF THE NAICA MINE (CHIHUAHUA, MEXICO)

The Naica mining district is located in a semi-desertic region (100 km southeast of the city of Chihuahua), on the northwest flank of the Sierra de Naica which, together with Sierra En medio and El Monarca, is elevated above an extensive alluvial floodplain. It is located inside of a hydrological basin (sub basin Tortuguillas) with an extension of approximately 1.300 km^2^. This area is characterized by small “arroyos” which drain run off water originated in the surrounding mountain ranges (Sierra de Naica, En Medio, and El Monarca), towards alluvial plains, which in their lower parts form closed basins. During the raining season these basins host temporary lagoons (e.g., Chancaplea, Agua Zoquete, and El Soldado lagoons located at, respectively, the west, southwest, and south of the Naica Mine), and the potential excess of superficial water might feed a shallow phreatic layer. Around these intermittent lagoons, marsh zones are observed subject to floods. These flooding zones have a NW–SE orientation and are parallel to the regional fault systems, thus representing possible recharge sources (Villasuso, 2002).

The materials of the Naica drainage basin, which host the ore deposit, are of Albian–Cenomanian age and form a gentle antiform structure made of a sequence of limestones and marls. This sequence is cut by discontinuous, pre-ore quartz–feldspar dikes (Megaw et al., 1988), which are associated to a relatively shallow subhorizontal igneous intrusion that still generates a broad thermal anomaly in the southwest part of the region. Magnetometric studies have unveiled an igneous source at a depth of between 2.5 and 5 km, some 4 km south of Naica (Villasuso, 2002), while in 2007 a drilling close to the mine shaft met an igneous body about 1,140 m below the surface (Forti and Sanna, 2010).

The ore deposit is made of extensive tabular bodies (**Figure 1**) and is related to hydrothermal flows induced by Tertiary dykes forming one of the largest chimney-manto (skarn) Ag–Pb–Zn deposits of Mexico (Erwood et al., 1979; Megaw et al., 1988; Palacios et al., 1991; Alva-Valdivia et al., 2003). Its ore mineralogy is represented mainly by early pyrite, galena, sphalerite, chalcopyrite, pyrrhotite, magnetite, rutile, and fluorite. The main ore-forming process was dated at 30.2–26.0 Ma (Alva-Valdivia et al., 2003, and references therein) and the early conditions of ore deposition were estimated to be about 400–500°C and 100–270 MPa (Megaw et al., 1988; Palacios et al., 1991). During a later stage, when the thermal fluids got colder, quartz, anhydrite, and calcite formed veins within the ore bodies (Stone, 1959). Mine activities have reached ca. 890 m below the mine entrance (level 0 at 1,385 masl), and they are some 760 m below the original groundwater level which was at -130 m (1,255 masl). To maintain the mine galleries water free a dewatering of about 1 m^3^/s is required.

As mentioned above, the Naica area is still under a mild thermal anomaly and water springing in the mine galleries has a temperature ranging from 50 to 60°C (Garcia-Ruiz et al., 2007). Two different fault sets are the main structural controls for hydrothermal circulation and therefore for the location of the mineral deposits. The most important of them are the Gibraltar, Naica, and Montaña faults. These structures still control the thermal water flow within the Naica anticline: almost all the water springing in the deep mine galleries comes from fractures related to these faults. Their important role in water circulation is also confirmed by the fact that karst caves have developed in their vicinity. These hypogenic caves (i.e., deep karst cavities that develop from fractured controlled flow of ground water within confided aquifers) formed well after the ore-forming event (Garofalo et al., 2010) and some of them exhibit impressive deposits of very large selenite crystals. For example, along a segment of the Naica fault several such caves have been discovered: Cave of crystals, Ojo de la Reina, and Cueva de las Velas. These caves developed completely isolated from the surface, under hundreds of meters of hydraulic head from the regional aquifer. Partial or effective confinement of this aquifer was achieved by the reduction of rock porosity from the precipitation of the hydrothermal minerals, and by the presence of shale levels (Benevides formation, **Figure 1**). The most typical cave mineralogy phases are gypsum, celestine, anglesite, jarosite, and goethite.

A detailed study of the geochemical and physicochemical characteristics of the thermal aquifer evidenced a novel formation mechanism of these giant crystals (Garcia-Ruiz et al., 2007) which is based upon the gypsum-anhydrite solubility disequilibrium. At 58°C the gypsum and anhydrite solubilities are the same (Hardie, 1967). At lower temperatures the solubility of gypsum becomes smaller than that of anhydrite. Therefore, below this temperature a solution saturated with respect to anhydrite is automatically super-saturated with respect to gypsum, thus inducing the deposition of gypsum and undersaturation with respect to anhydrite. Hence, the development of a few large crystals instead of many small ones is due to the fact that the temperature drop was extremely slow (data from fluid inclusions indicate that the giant crystals developed in a temperature range between 55 and 58°C (Krueger et al., 2013); which lead to reduced nucleation (Garcia-Ruiz et al., 2007) and a very low growth rate (Van Driessche et al., 2011).
